# Identification of a New Variant in NLRP3 Gene by Whole Exome Sequencing in a Patient with Cryopyrin-Associated Periodic Syndrome

**DOI:** 10.1155/2021/2023119

**Published:** 2021-08-16

**Authors:** Mahdieh Vahedi, Nima Parvaneh, Saeedeh Vahedi, Mohammad Shahrooei, Vahid Ziaee

**Affiliations:** ^1^Children's Medical Center, Pediatrics Center of Excellence, Tehran, Iran; ^2^Department of Pediatrics, Tehran University of Medical Sciences, Tehran, Iran; ^3^Department of Microbiology and Immunology, Laboratory of Clinical Bacteriology and Mycology, KU Leuven, Leuven, Belgium; ^4^Pediatric Rheumatology Research Group, Rheumatology Research Center, Tehran University of Medical Sciences, Tehran, Iran; ^5^Pediatric Rheumatology Society of Iran, Tehran, Iran

## Abstract

**Background:**

NLRP3 gene is located in chromosome 1 and encodes a pyrin-like protein. Mutations in this gene are associated with an autoinflammatory disease, called cryopyrin-associated periodic syndrome (CAPS). *Case Presentation*. We report a 1-year-old boy who had recurrent urticarial rash since birth and joint pain and swelling. He had a missense mutation c.G1060 T (p.A354S) in exon 5 of the NLRP3 gene which was detected by whole exome sequencing.

**Conclusion:**

A novel variant was found in the NLRP3 gene which has not been reported by now.

## 1. Background

Cryopyrin-associated periodic syndrome (CAPS) is a rare autoinflammatory disorder which inherited as an autosomal dominant condition. It includes three clinically overlapping syndromes with a spectrum of severity of symptoms: (1) familial cold autoinflammatory syndrome (FCAS), (2) the Muckle–Wells syndrome (MWS), and (3) the chronic infantile neurological, cutaneous, and articular syndrome (CINCA) [1]. Mutations in NLRP3 which is located on long arm of chromosome 1 are causal for CAPS. It encodes a protein comprising a pyrin domain, a nucleotide-binding site (NBS) domain, and a leucine-rich repeat (LRR) motif. The protein is a member of inflammasome complex which has a major role in innate immunity. This complex is made up of NLRP3, PYCARD, and CASP1. In response to a pathogen or other damage-associated signals, NLRP3 initiates assembly of this complex. After caspase-1 activation, proinflammatory cytokines IL-1B and IL-18 are secreted. There are about 100 pathogenic mutations in NLRP3 gene in patients with CAPS [2–4]. The relationship between NLRP3 mutations and these diseases was identified in early 2000s.

We identified a case of CAPS, who had recurrent urticarial rash, high grade fever, and chronic joint involvement. His symptoms did not response to therapy, and whole exome sequencing reported new variant in the NLRP3 gene.

Whole exome sequencing is a tool for identifying genes which can cause a disease. It is also useful for diagnosis in the clinic.

## 2. Case Presentation

**Figure 1:**
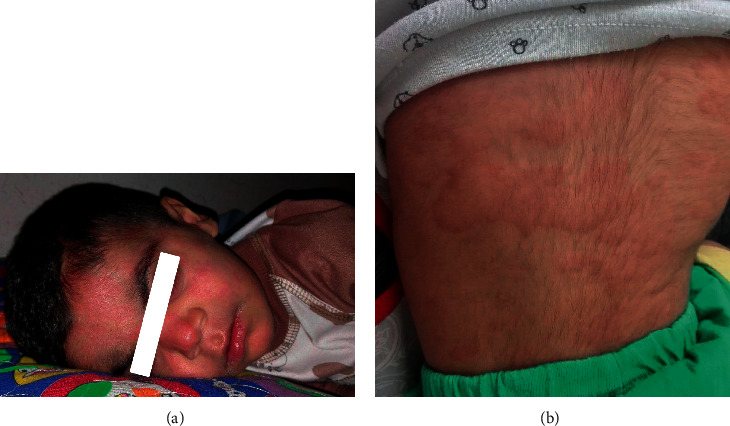
Urticaria-like rash on the face (a) and trunk (b).

A 1-year-old boy was brought to our clinic with joint pain and swelling in the left ankle and both knees for more than 14 days. He had recurrent urticarial rash since birth, and this rash was not triggered by cold or other physical stimulus (Figure 1). His urticarial rash often presented with high grade fever. His parents were not blood relatives. On examination, neurodevelopmental evaluation was normal, but his weight and height were below the 3^rd^ percentile for age. He had significant synovial hypertrophy in both knees. The other parts of physical examinations were normal. Laboratory findings showed leukocytosis, anemia, and thrombocytosis. Chest X-ray and abdominal ultrasonography were normal. Echocardiography showed normal ventricular function and no pericardial effusion. Due to prolonged fever, recurrent urticarial rash, arthritis, and increase in acute phase reactants, the diagnosis of autoinflammatory disorders, especially CAPS was suggested for him. The genetic test was performed, and whole exome sequencing identified heterozygous mutation of NLRP3 gene, and it was de novo mutation.

## 3. Exome Sequencing

Genomic DNA was extracted from peripheral blood using the MG Blood Genomic DNA Extraction Miniprep (CancerRop) according to the manufacturer's instruction, and whole exome sequencing was performed for the patient. The library was enriched with an Agilent SureSelect Human All Exon V6 kit (Agilent Technologies Inc., USA) and sequenced with 100× coverage on Novaseq6000 platform (Illumina Inc., USA) to generate 150 bp paired-end reads.

After passing quality control, raw data were aligned against human reference genome (hg19, NCBI Build 37) by BWA (Burrows–Wheeler Aligner) software (http://www.bio-bwa.sourceforge.net). Picard was utilized to remark duplicates. Variant calling was performed using GATK (Genome Analysis Toolkit) (http://www.broadinstitute.org/gatk/), and variant annotation was performed by ANNOVAR online software (http://www.openbioinformatics.org/annovar/).

**Figure 2:**
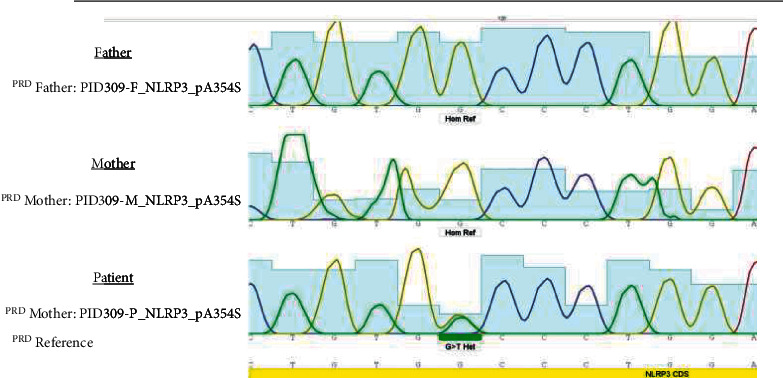
Sequence electropherogram of NLRP3 (c.G1060 T). The parents were homozygous for wild-type allele, but the patient was heterozygote for mutant allele.

The candidate variants identified by whole exome sequencing were confirmed by Sanger sequencing (Figure 2).

## 4. Discussion

The symptoms of cryopyrin-associated periodic fever syndromes are variable, and they are present with a broad range of clinical manifestations. Inheritance of CAPS is usually autosomal dominant. The disease has a spectrum of symptoms in different generations. The severity of symptoms increases with age. For example, in first generation, the disease may present as amyloidosis and renal failure, but in younger children, urticaria and fever can be observed [5–8].

NLRP3 gene is located on chromosome 1q44. It has 11 exons and multiple alternatively spliced transcript variants. The gene encodes a pyrin-like protein. NLRP3 is a member of the NLRP3 inflammasome complex. This protein is an activator of the NF-kappa B signaling pathway, so it has a role in inflammation and the immune response. Mutations in this gene are associated with an autoinflammatory disease known as CAPS (cryopyrin-associated periodic syndrome). Gain of function mutations in CAPS patients leads to hyperactive cryopyrin inflammasome, increased myeloid cell-derived proinflammatory cytokine release, and systemic and tissue inflammation leading to disease symptoms. Most mutations in patients with CAPS are located in exon 3 [9, 10].

The relationship between genetic alterations in the NLRP3 gene and CAPS was reported first in 2001 [11].

In this study, we performed whole exome sequencing in a patient with a diagnosis of CAPS. The results showed a new variant in exon 5 of NLRP3 (NM-001079821.3: c.G1060 T, p.A354S). In silico prediction of pathogenicity was performed with functional prediction programs that included BayesDel_addAF, DANN, EIGEN, FATHMM-MKL, CADD, and MutationTaster. So, based on American College of Medical Genetics and Genomics guideline for variant classification, this variant is pathogenic by using the VarSome and InterVar databases [12]. The variant was predicted to be deleterious (CADD score 37).

The patient was heterozygous for this variant. The mutation was not present in either parent, indicating that the mutation was a de novo or germline mutation. rs180177503 is a single nucleotide variant that can cause c.1060 G > A missense mutation based on the NCBI database. The reported variant in this study is a c.1060 G > T mutation and has not reported yet. Given, prenatal diagnosis was recommended to his parents for next pregnancies. All of the NLRP3 exons were sequenced by Sanger sequencing for this patient and his parents, and there was no other pathogenic or likely pathogenic variant in NLRP3.

## 5. Conclusion

Our study identified a novel variant in NLRP3 gene which was associated with CAPS and increased the pathogenic variant spectrum for this gene. Also, more functional studies are important to develop new approaches for treating CAPS.

## Data Availability

The data used to support the findings of this study are available from the corresponding author upon request.
